# How Accurate Are Electronic Monitoring Devices? A Laboratory Study Testing Two Devices to Measure Medication Adherence

**DOI:** 10.3390/s100301652

**Published:** 2010-03-02

**Authors:** Leentje De Bleser, Sabina De Geest, Sofie Vandenbroeck, Johan Vanhaecke, Fabienne Dobbels

**Affiliations:** 1 Centre for Health Services and Nursing Research, Katholieke Universiteit Leuven, Kapucijnenvoer 35 box 7001, B-3000 Leuven, Belgium; E-Mails: Leentje.DeBleser@med.kuleuven.be (L.D.B.);Sofie.Vandenbroeck@med.kuleuven.be (S.V.); 2 Institute of Nursing Science, University of Basel, Basel, Switzerland; E-Mail: Sabina.DeGeest@unibas.ch; 3 Heart Transplantation Program, University Hospitals of Leuven, Leuven, Belgium; E-Mail: johan.vanhaecke@uz.kuleuven.be

**Keywords:** electronic monitoring, accuracy, laboratory study, adherence behaviour

## Abstract

In a prospective descriptive laboratory study, 25 Helping Hand^™^ (HH) (10 without and 15 with reminder system) and 50 Medication Event Monitoring Systems (MEMS) (25 with 18-month and 25 with 2-year battery life) were manipulated twice daily following a predefined protocol during 3 consecutive weeks. Accuracy was determined using the fixed manipulation scheme as the reference. Perfect functioning (*i.e.*, total absence of missing registrations and/or overregistrations) was observed in 70% of the HH without, 87% of the HH with reminder, 20% MEMS with 18 months, and 100% with 2-year battery life respectively.

## 1. Background

Non-adherence is a prevalent problem in chronically ill populations and it may result in poor clinical and economic outcomes [1]. Measurement of medication non-adherence is crucial to identify patients at risk for poor outcomes and to evaluate adherence-enhancing interventions. Several assessment methods exist, but all have inherent weaknesses [2]. Electronic monitoring (EM) is often promoted as “the gold standard”, as it is the only assessment tool allowing continuous monitoring over time, generating information regarding taking and timing of drug intake. It is also capable of capturing minimal deviations from the prescribed regimen, which is an asset particularly for populations (e.g., transplant or HIV) in which minimal deviations from dosing schedule are already sufficient to result in poor clinical outcomes [3,4].

**Figure 1.**
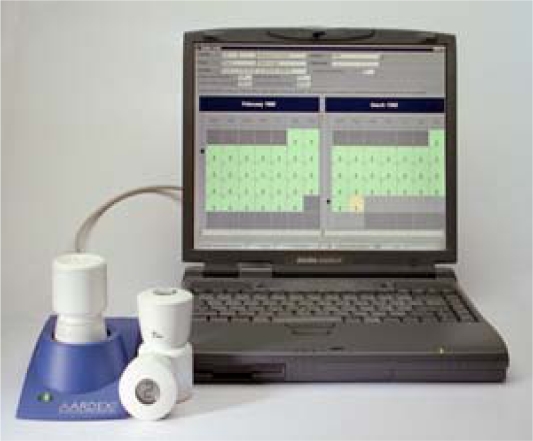
The Medication Electronic Monitoring System (MEMS) (Aardex, Switzerland).

To date, the Medication Event Monitoring System (MEMS, Aardex, CH) has been most frequently used. It consists of a pill bottle, fitted with a cap, containing a microelectronic circuit, that registers the date and time of opening of the bottle (see Figure 1). Yet, the MEMS system has important drawbacks: it may lead to practical (e.g., the pill bottle is rather large) or confidentiality issues (e.g., HIV patients have addressed privacy issues) [4]. Besides, based on the safety regulations from the pharmaceutical companies, some medication tablets (e.g., immunosuppressants) need to stay in the blister until actual ingestion to avoid changes in stability of the drugs due to exposure to moisture, air, light, and microbiological cross-contamination [5]. In order to fit in the EM medication container, the blister needs to be cut in individual packaged pills.

In recent years, other electronic monitoring devices have been developed. Bang & Olufsen, Medicom (Denmark) developed the Helping Hand^™^ Data Capturing (HH), an electronic monitoring tool with similar functions compared to the MEMS, but suitable for blister packages. This new device seems very promising to cover e.g., privacy problems because it is smaller and easy to hide compared to e.g., MEMS. Furthermore, the blister does not need to be cut anymore to fit into the device. Besides, the reminder system consists of Light Emitting Diodes (LED) reminders providing feedback to the user regarding their medication behavior within the previous week via a patented traffic-light color-codes system. A red flashing lamp indicates major NA (“alarm”), orange indicates minor NA (“you missed doses”) and green means perfect adherence (“everything is fine”). The acoustic function yields a beeping signal at the time of the scheduled medication intake.

Most of EM devices are used in patient populations without prior formal testing by an independent researcher to determine if the devices function correctly, and whether the data stored in the device reflect the actual behavior performed by the patient [6]. The accuracy of all electronic monitoring devices should be tested before they can be implemented in clinical studies or daily clinical practice. Elements that can be considered in the testing can be derived from a recently developed conceptual framework [6]. *Accuracy* is the degree of veracity or conformity of a measured quantity to its true value. The accuracy of a measurement process is usually tested in a semi-laboratory setting, in which the devices under study are manipulated by a researcher following a predefined, standardized protocol. The latter is used as the reference standard against which deviations in terms of underregistration or overregistration of a given behavior are compared. No studies have been published so far, testing the accuracy of electronic monitoring devices. The primary aim of this study was to test the accuracy of the Helping Hand^™^ and the MEMS.

## 2. Methodology

### 2.1. Design

The accuracy of a measurement process is usually established by repeatedly measuring a traceable reference standard. This can best be done in a situation in which several devices are manipulated in standardized circumstances following a predefined schedule that is considered to be the gold standard. Therefore, to test the accuracy of the HH and the MEMS, a prospective sequential descriptive laboratory study was executed.

### 2.2. Sample and Setting

In total, we tested 25 HH devices and 50 MEMS devices.

#### 2.2.1. Helping Hand^™^

The devices tested in this study were intended to be used in an adherence intervention study. Twenty-five HH devices were randomly selected from a batch of 831 devices by an independent researcher using a random numbers table: 10 HH monitoring systems (randomly selected from 406 systems with monitoring function only—*i.e.*, 2.5% of the devices with monitoring function only) and 15 HH devices (randomly selected from the 425 devices that not only have the electronic monitoring function, but also an acoustic reminder and visual feedback function—*i.e.*, 3.5% of the devices).

#### 2.2.2. Medication Event Monitoring System

For the MEMS devices, two sets with a different battery life were selected. A first set consisted of 25 devices that had a battery life of 18 months and had been already activated at the time of delivery to our research unit. A second set of 25 MEMS devices had a battery life of 2 years, but needed to be activated by the researchers. These were randomly selected from 50 devices purchased for use in a clinical study. Both types were MEMS 6 Smart Caps. The purpose of the present study was not revealed to the manufacturers.

### 2.3. Variables and Measurement

A three week manipulation schedule was developed as a reference. This is considered to be the ‘gold standard’. In order to mimic the twice daily regimen of medication taking, every device was manipulated twice daily with exactly twelve hours between two manipulations. To imitate patients that take extra medications or take their medication on variable hours, on three randomly chosen days (day 9, 12 and 18) there were four manipulations (every 6 hours) in each day. The researcher noted the following information for the Helping Hand^™^ testing on a standardized sheet: date; exact time of manipulation of the system (*i.e.*, removal of the blister); color of the visual feedback lamp at the time of the scheduled manipulation (in the devices with this function only); and time that the reminder function started beeping (in the devices with this function only). With respect to the MEMS, the following information was noted: date; and exact time of manipulation of the system (*i.e.*, removal of the cap).

### 2.4. Procedure

After having randomly selected the devices, each of the HH monitoring systems were labeled with a number (1–15 for the electronic monitoring devices with reminder and feedback function, and 16–25 for the devices with monitoring only). At the time stated in the manipulation protocol, all devices were manipulated. More specifically, for the HH devices, a manipulation was defined as “removing the blister out of the device, waiting until you can hear a beeping signal and after the signal reinserting the blister into the device”. The devices were manipulated as if there was a twice daily medication regimen with an intake at 8.30 o’clock in the morning and 8.30 o’clock in the evening. All the devices were always manipulated in the same order, starting with device 1, afterwards device 2, *etc.* with 30-second intervals between 2 consecutive devices. The exact date and time of the manipulation were always registered using the same watch, which was synchronized online with the atom clock. After each manipulation, the date and time were noted on a special sheet developed for the purpose of this study. With respect to the HH devices with an acoustic reminder, the starting time of the beeping signal was also registered. For the MEMS devices the same procedure was used. A manipulation was defined as: removing the cap of the bottle, waiting for 3 seconds, and closing the bottle with the cap. Quality control was performed by a second researcher by unannounced observation at randomly selected time points spread over the 3-week time period.

### 2.5. Statistical Analysis

After the 3-week data collection period, the stored data were downloaded on a computer. For the HH devices, the HelpView software was used. For the MEMS devices, the powerview program was used. For data analysis, Office XP Excel was used. The date and time stamps indicated on the printouts of the HH and MEMS were compared with the date and times indicated on the manipulation scheme from the researcher (*i.e.*, the reference value). Data analysis was performed at 2 levels, *i.e.*, “event level” (based on the number of manipulations) and “device level”. *On the manipulation event level*, the following calculations were made: (1) for HH without reminder system: % missing and overregistrations of devices per total number of events (420 = 10 devices × 42 manipulations), (2) for the HH with reminder system: % missing and overregistrations of devices per total number of events (630 = 15 devices × 42 manipulations), (3) for MEMS with 18-month battery life: % missing and overregistrations per total number of events (1,050 = 25 devices × 42 manipulations) and (4) for MEMS with 2-year battery life: % missing and overregistrations per total number of events (1,050 = 25 devices × 42 manipulations).

On *the Electronic Monitoring device level,* we calculated: (1) the % missing and overregistrations of HH (N = 25 = 10 basic devices and 15 reminder devices) and MEMS devices (N = 50 = 25 devices with 18-month battery life and 25 devices with 2-year battery life); and (2) number of devices with 100% correct registrations (total number of devices = 75).

The MEMS is programmed to ignore extra registrations within 15 minutes after the initial registration. In order to enhance the comparability of the results of the MEMS and HH, we also deleted extra registrations in the HH that happened within 15 minutes after the initial registration. Hence, these registrations were not categorized as an overregistration.

## 3. Results

In total, 3,150 manipulations were conducted. In Table 1, the results of the accuracy of the Helping Hand™ and the MEMS devices are described. At ‘event level’, there were 5/420 (1.20%) and 1/630 (0.16%) missing registrations for the HH devices without reminder and HH devices with reminder, respectively. For the MEMS devices, there were 33/1050 (3.14%) for MEMS with 18-month battery life, but no missing registrations for MEMS with 2-year battery. Only a few overregistrations were recorded: 2/420 (0.48%) in the HH without reminder and 1/630 (0.16%) in the HH with reminder. There were no overregistrations in either MEMS group.

At ‘device level’, HH devices without reminder (N = 10) performed worse compared to HH with reminder (N = 15). Three (30%) HH without reminder had missing values in comparison with 1/15 (6.67%) of the HH with reminder. The number of missing registrations of a device ranged between 1 and 2. The MEMS with short battery life performed worse compared to the MEMS devices with 2-year battery life. Twenty (80%) MEMS with short battery life had missing values in comparison to 0.0% in the other MEMS system. Overregistrations were recorded in 2/10 (20%) of HH without reminder and in 1/15 (6.67%) of HH with reminder. No overregistrations were recorded with the MEMS systems. Overall, 7 out of 10 (70%) HH devices without reminder functioned perfectly. For the HH devices with reminder system this was 13/15 (87%). For the MEMS devices, five (20.00%) of the devices with 18-month battery functioned perfectly. For the MEMS devices with 2-year battery life this was 100.0%.

## 4. Discussion and Conclusion

To our knowledge, this was the first independent study assessing the accuracy of MEMS and HH devices using data of a fixed manipulation scheme as reference standard. Actually, both systems show some measurement error ranging between 0.16% and 1.20% at the event level for the HH and between 0% and 3.14% for the MEMS. At the device level, 70%–87% of HH functioned 100% correctly. This ranged between 20%–100% for the MEMS. The results suggest that battery life plays an important role in the accuracy of the MEMS: more specifically, a battery life of 2 years increases the likelihood of 100% correct functioning. For the HH, under- and overregistrations occur. The results of this study confirm Denhaerynck’s [6] advice to test the performance of electronic monitoring technology before use in clinical studies is imperative.

The reasons for under- and over-registrations remain unclear. For the MEMS, we used the same types of caps, *i.e.*, 6 SMART CAP. We also ordered the devices with shorter battery life in November; hence weather conditions during transportation could have played a role. Both Aardex and Bang & Olufsen Medicom already performed extensive tests on their products. More specifically, Aardex performed tests on the water-resistance, temperature resistance (from 0 °C to 60 °C), shock resistance, child resistance, battery life, and long term (1 year) daily use of the MEMS, to ensure correct recording of openings and closings of the MEMS monitor. Aardex reports a failure rate below 0.5% [7]. The MEMS are CE-marked and have successfully passed the Electromagnetic Compatibility Tests run by Montena, a test laboratory accredited by Metas, the Swiss Federal Office of Metrology and Accreditation. Bang & Olufsen, Medicom also tested the quality of the devices in a stable laboratory situation where they are exposed to extreme circumstances such as heat, water and shocks. Given that both manufacturers expose their devices to extreme temperatures, decreases the likelihood that weather conditions during transportation have played a role. Our study focused more on the accuracy of the devices when manipulated in order to mimic a patient’s medication intake in stable circumstances, *i.e.*, without external influences such as heat, water, shocks, specific patient characteristics such as tremor, or forgetfulness. Given that we observed measurement errors, albeit infrequently, and irrespective of the reason for this, it is recommended that all electronic devices undergo accuracy testing before using them in clinical studies or daily life.

Researchers and clinicians who use either the HH or MEMS should be aware of the possibility of measurement errors. Indeed, an inadequate measurement of non-adherence due to inaccurate EM devices can have clinical consequences, particularly in diseases in which minor deviations from prescribed dosing and timing of drug administration are enough to increase the risk of poor clinical outcomes [3,8]. In transplantation, for instance, taking less than 98% of the immunosuppressive doses results in significantly more graft losses [9]. Similarly, in HIV, taking less than 95% of the antiretroviral drugs could be harmful in terms of suboptimal viral suppression and development of resistance [4]. If a patient is wrongfully labeled as adherent because of overregistration, a clinician may think a patient experiences no effect of a given treatment, and may subsequently change the dose or the type of medication. On the other hand, if there are underregistrations, patients will be wrongly categorized as non-adherent, yielding patients to be blamed for their presumed non-adherence, whereas patients themselves become confused about their actual medication behavior. Therefore, very sensitive measurement tools that function 100% correctly are needed to capture minor deviations in medication intake.

## 5. Methodological Issues

Although this is an important study to identify the accuracy of the MEMS and the Helping Hand™ devices, there are some methodological issues that we need to address. First, since the study protocol is performed by a single researcher, it is important that the correctness of carrying out the protocol is checked by an additional independent researcher. In the present study, a second researcher, who was familiar with the manipulation protocol, performed a quality control by observing the researcher at randomly selected time points spread over the 3-week time period. In addition, this independent researcher replicated the study in exactly the same manner. The results obtained by this second researcher (results not reported) were comparable to those reported in this article, indicating that there is no influence from the researcher who executed the study. In addition, during the analysis, all data were also checked by this second researcher, to minimize error while importing the data from the reference standard and the registered manipulations to the computer. She also particularly checked if the bottles remained open long enough before closing them (*i.e.*, at least 3 seconds according to the manufacturer), or whether the blister was removed from the HH long enough to enable a registration. Hence speed of manipulation of the devices did not play a role in the results.

Second, we used a three weeks testing period. This could be considered as relatively short. We decided to monitor during three weeks because of practical reasons (day and night observations). Indeed, executing such a laboratory study and analyzing the data are very time consuming. Studies including a longer testing period are advocated to get an overview of the influence of time on the accuracy of the EM device.

Third, a limited number of devices were tested. We tried to avoid possible bias by randomly selecting the devices from the available batches. In the future, accuracy studies could be replicated using a larger number of the different MEMS and Helping Hand™ devices.

Fourth, we tested MEMS and HH in stable laboratory circumstances. However, it is important that the accuracy of EM devices is also tested when used in daily life. For example, carrying the Helping Hand™ in a purse may evoke a medication intake registration, recorded by movements during walking and not by a medication intake registration event.

Fifth, accuracy is only one crucial aspect of electronic monitoring. In addition to the objective tests, further research specifically focusing on the subjective dimension, *i.e.*, the patients’ perspective, is important. As patients are the end users, EM devices have to be evaluated with respect to user performance, satisfaction and acceptability. Therefore, we also conducted a study to evaluate the user experiences with respect to the HH. The results of this study will be reported separately.

## 6. Conclusions

This was the first independent study assessing reliability of MEMS and HH using data of a fixed manipulation scheme as reference standard. We observed missing registrations in 1.20% and 0.16% of HH without and with reminder, respectively; and in 3.14% and 0% of MEMS with 18 months and with 2-year battery life, respectively. Overregistrations were recorded in 0.48% of HH without reminder and in 0.16% of HH with reminder. No overregistrations occurred in MEMS. Perfect functioning was observed in 70% of the HH without, 87% of the HH with reminder, 20% MEMS with 18 months, and 100% with 2-year battery life respectively. We recommend that the accuracy of all EM systems is formally tested before using them in research or clinical settings.

## Figures and Tables

**Figure 2.**
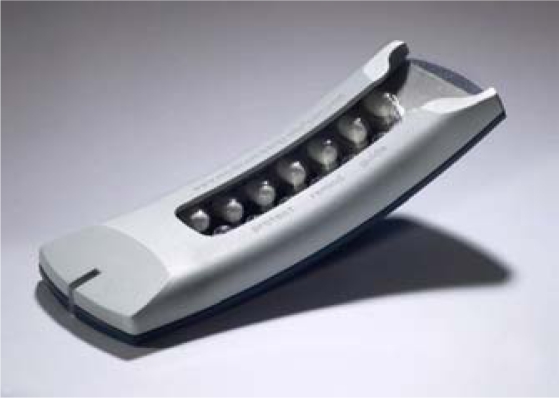
The Helping Hand™ (HH) (Bang & Olufsen Medicom, Denmark).

**Table 1.** Results of the accuracy of the Helping Hand™ and the MEMS.

	**HH without reminder**	**HH with reminder**	**MEMS 18 month battery**	**MEMS 2 year battery**
**Event level**				
Missing registrations	5/420 (1.20%)	1/630 (0.16%)	33/1050 (3.14%)	0/1050 (0%)
Overregistrations	2/420 (0.48%)	1/630 (0.16%)	0/1050 (0%)	0/1050 (0%)
**Device level**				
Missing registrations	3/10 (30%)	1/15 (6.67%)	5/25 (20%)	0/25 (0%)
Overregistrations	2/10 (20%)	1/15 (6.67%)	0/25 (0%)	0/25 (0%)
100% correct functioning	7/10 (70%)	13/15 (87%)	20/25 (80%)	25/25 (100%)
